# Conservatively Treated Incidental Aneurysm of the Distal Left Main Coronary Artery: Detection by Coronary Angiography and Noninvasive Followup Using Coronary Computed Tomography Angiography

**DOI:** 10.1155/2012/547318

**Published:** 2012-05-27

**Authors:** Nina P. Hofmann, Hassan Abdel-Aty, Stefan Siebert, Hugo A. Katus, Grigorios Korosoglou

**Affiliations:** ^1^Department of Cardiology, University of Heidelberg, 69120 Heidelberg, Germany; ^2^Department of Radiology, University of Heidelberg, 69120 Heidelberg, Germany

## Abstract

Coronary artery aneurysms are relatively rare and commonly associated with significant coronary artery disease (CAD), inflammatory diseases (Kawasaki syndrome, infection), or iatrogenic complications. Herein, we report an unusual case of an incidental coronary aneurysm of the left main artery in a patient without specific clinical symptoms of myocardial ischemia or systemic inflammation and without angiographically significant CAD. Angiographic images are provided, acquired during cardiac catheterization, as well as coronary computed tomography angiography (CCTA) images obtained at 1 year of followup.

## 1. Case Report

 A 74-year-old male patient with history of premature ventricular beats documented as LOWN class IVa since 2006 was admitted to our department in November 2010 with vertigo, nausea, and presyncope due to symptomatic bradycardia with pauses of up to 3.5 seconds. Physical examination revealed no pathologic cardiopulmonary findings and laboratory tests, including kidney function (serum creatinine 0.97 mg/dL), C-reactive protein (<2 mg/L), and leucocytes (7.8/nL), were normal. Furthermore, high-sensitive Troponin T (8 pg/mL) was normal, and diabetes mellitus (HbA1c of 5.8% and fasting glucose of 98 mg/dL) was excluded.

Due to the symptomatic bradycardia the patient was implanted with a cardiac pacemaker. Furthermore, he was scheduled for cardiac catheterization due to global mildly impaired left ventricular function by echocardiography (ejection fraction of 45%). Specific symptoms of ischemic heart disease as exertional angina or dyspnoea were not present.

**Figure 1:**
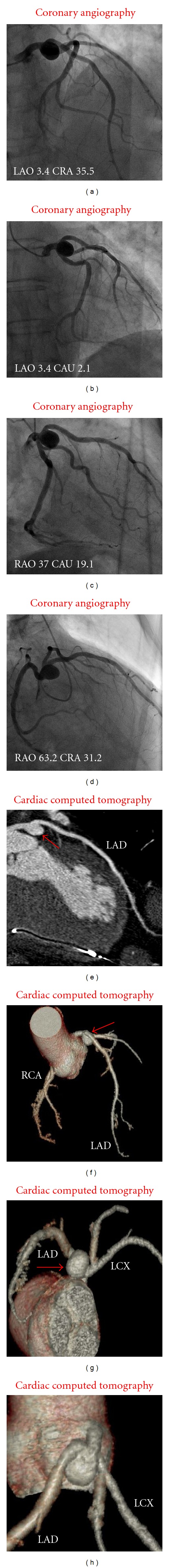
Images of the coronary aneurysm by coronary angiography ((a)–(d)) and cardiac computed tomography angiography ((e)–(h)). Red arrows point to the coronary aneurysm. LAD: left anterior descending, LCX: left circumflex coronary artery; LAO: left anterior oblique, and RAO: right anterior oblique projections.

 During coronary angiography a coronary aneurysm in the distal left main artery was observed with a diameter of 13 mm × 11 mm (Figures 1(a)–1(d)). Overall, no significant coronary atherosclerosis was noted except for discrete wall irregularities in the left anterior descending and left circumflex coronary artery. Left ventricular (LV) angiography confirmed mildly impaired function of the left ventricle compatible with an initial phase of dilated cardiomyopathy. Due to lack of symptoms a conservative treatment regime was decided for the coronary aneurysm and the patient was discharged medications including aspirin (100 mg/d), bisoprolol (5 mg/d), ramipril (7.5 mg/d) and pravastatin (20 mg/d) for treatment of mild coronary artery disease, arterial hypertension and dyslipidemia.

 At one year of followup the patient was scheduled for coronary computed tomography angiography (CCTA) in order to investigate if a size progression of the diagnosed aneurysm is present. Hereby, a 256-slice CCTA (Philips Best, Netherlands Healthcare) was performed, exhibiting a coronary aneurysm of stable location and form and without evidence of thrombosis or progression in size (constant diameter of 13 mm × 11 mm) (Figures 1(e)–1(h)).

## 2. Discussion

 Aneurysms of the coronary arteries are relatively rare and most commonly associated with significant atherosclerotic disease [1]. Other causes include Kawasaki disease, infection, dissection, congenital [2], or postiatrogenic reasons (i.e., during percutaneous interventions) [3]. Conversely, in our patient none of such inflammatory conditions were present, while he had only discrete signs of coronary artery disease by angiography and CCTA; that is, merely lumen irregularities of the left main, left anterior descending, and left circumflex coronary artery. However, the presence of a connective tissue disorder was not excluded by genetic testing.

Our case illustrates the role of non-invasive imaging in the diagnostic workup and followup of these cases. Although the aneurysm was typically detected with coronary angiography, CCTA better illustrated the geometry and morphology of the lesion as well as its relation to neighbouring anatomical structures, as the aortic root. Such information would be important if surgical treatment is considered.

Thrombosis and embolisation on the other hand are potential complications of coronary aneurysms, resulting in myocardial infarction or rupture with subsequent pericardial tamponade. The long-time prognosis and optimal treatment are not fully established so far. Symptomatic aneurysms, causing angina pectoris and dyspnea, are preferably treated with bypass surgery with or without resection of the aneurysm [4]. Some surgeons empirically even recommend this strategy in the lack of anginal symptoms in case of large and saccular aneurysms with higher risk of rupture (at least three times larger than the corresponding coronary diameter) [5]. However, this approach cannot be supported by prospective clinical data. In addition, the use of oral anticoagulation for the conservative treatment of coronary aneurysms in symptomatic patients remains controversial [6, 7]. In this regard, supporters of anticoagulation treatment point to the slow or turbulent blood flow within aneurysm formation and to injured endothelium, both potential contributors of thrombus formation [8].

Furthermore, the exact mechanisms of aneurysm formation are not fully understood. In this regard, current data indicate the role of inflammatory mechanisms for the formation of aneurysms for instance in Kawasaki disease. Hereby, the role of a follistatin-like protein 1 has been discussed previously [9]. Drug-eluting balloons [10] and drug-eluting stents, on the other hand, were reported to be associated with the formation of coronary aneurysms [11] possibly due to activation of leucocyte surface adhesion molecules, which act as modulators of local inflammation [12].

In our case, and because our patient had no specific symptoms of CAD, we decided to follow a conservative treatment regime including antiplatelet-therapy instead of oral anticoagulation. A noninvasive followup of the coronary aneurysm by CCTA may be considered in such patients without symptoms, as it allows for noninvasive visualization of such lesions and the detection of potential thrombotic complications. Due to the implementation of modern dose reduction strategies, which allow for diagnostic image acquisition with a resultant radiation exposure of ~1.0 mSv, CCTA can in the meantime be performed if required serially in such patients [13, 14].

## References

[1] Oliveros RA, Falsetti HL, Carroll RJ, Heinle RA, Ryan GF (1974). Atherosclerotic coronary artery aneurysm. Report of five cases and review of literature. *Archives of Internal Medicine*.

[2] Dundar C, Tigen K, Pala S, Sasmazel A, Kirma C (2011). Congenital left main coronary artery aneurysm. *Cardiology Journal*.

[3] Ruiz-Nodar JM, Valencia J, Pineda J (2007). Coronary aneurysms after drug-eluting stents implantation. *European Heart Journal*.

[4] Wells TA, Peebles CR, Gray HG (2008). Giant left anterior descending coronary artery aneurysm. *International Journal of Cardiology*.

[5] Ebert PA, Peter RH, Gunnells JC, Sabiston DC (1971). Resecting and grafting of coronary artery aneurysm. *Circulation*.

[6] Moreno NFM, Gonçalo L, Castro A (2011). Left main coronary artery aneurysm. *Revista Portuguesa de Cardiologia*.

[7] Manlhiot C, Brandão LR, Somji Z (2010). Long-term anticoagulation in Kawasaki disease: initial use of low molecular weight heparin is a viable option for patients with severe coronary artery abnormalities. *Pediatric Cardiology*.

[8] Grigorov V (2009). Invasive and anticoagulant treatment for coronary ectasia: a single operator’s experience in a tertiary hospital in South Africa. *Cardiovascular Journal of Africa*.

[9] Gorelik M, Wilson DC, Cloonan YK, Shulman ST, Hirsch R Plasma follistatin-like protein 1 is elevated in Kawasaki disease and may predict coronary Artery aneurysm formation.

[10] Vassilev D, Hazan M, Dean L Aneurysm formation after drug-eluting balloon treatment of drug-eluting in-stent restenosis: first case report.

[11] Akin I, Kische S, Rehders TC (2011). Very late coronary aneurysm formation with subsequent stent thrombosis secondary to drug-eluting stent. *Chinese Medical Journal*.

[12] Adiloglu AK, Ocal A, Tas T, Onal S, Kapan S, Aridogan B (2011). Increased expression of CD11a and CD45 on leukocytes and decreased serum TNF-alpha levels in patients with isolated coronary artery ectasia. *Clinical Laboratory*.

[13] Hosch W, Stiller W, Mueller D Reduction of radiation exposure and improvement of image quality with BMI-adapted prospective cardiac computed tomography and iterative reconstruction.

[14] Hosch W, Heye T, Schulz F (2010). Image quality and radiation dose in 256-slice cardiac computed tomography: comparison of prospective versus retrospective image acquisition protocols. *European Journal of Radiology*.

